# How socio-ecological factors influence the differentiation of social relationships: an integrated conceptual framework

**DOI:** 10.1098/rsbl.2020.0384

**Published:** 2020-09-16

**Authors:** Liza R. Moscovice, Cédric Sueur, Filippo Aureli

**Affiliations:** 1 Institute of behavioural physiology, Leibniz Institute for Farm Animal Biology, Dummerstorf, Germany; 2 Department of Ecology, Physiology and Ethology, Université de Strasbourg, CNRS, IPHC, UMR 7178, F-67000 Strasbourg, France; 3 Institut Universitaire de France, Paris, France; 4 Instituto de Neuroetología, Universidad Veracruzana, Xalapa, Mexico; 5 Research Centre in Evolutionary Anthropology and Palaeoecology, Liverpool John Moores University, Liverpool, UK

**Keywords:** group living, social complexity, competition, cooperation, information sharing, pathogen transmission

## Abstract

The extent of differentiation of social relationships within groups is a means to assess social complexity, with greater differentiation indicating greater social complexity. Socio-ecological factors are likely to influence social complexity, but no attempt has been made to explain the differentiation of social relationships using multiple socio-ecological factors. Here, we propose a conceptual framework based on four components underlying multiple socio-ecological factors that influence the differentiation of social relationships: the extent of within-group contest competition to access resources, the extent to which individuals differ in their ability to provide a variety of services, the need for group-level cooperation and the constraints on social interactions. We use the framework to make predictions about the degree of relationship differentiation that can be expected within a group according to the cumulative contribution of multiple socio-ecological factors to each of the four components. The framework has broad applicability, since the four components are likely to be relevant to a wide range of animal taxa and to additional socio-ecological factors not explicitly dealt with here. Hence, the framework can be used as the basis for the development of novel and testable hypotheses about intra- and interspecific differences in relationship differentiation and social complexity.

## 1. Introduction

Group living is widespread across animal taxa [1]. One of its primary consequences is that group members have opportunities to interact with one another and form social relationships, which are characterized by the frequency, patterning and type (e.g. affiliative, aggressive) of social interactions that they exchange [2]. Variation in the frequency, patterning and types of interactions among group members determines the diversity of social relationships and the extent of relationship differentiation within a group [3].

Socio-ecological models (SEMs) aim to explain how various social and ecological factors influence the nature of social relationships within groups (i.e. demographically stable subsets of conspecifics who interact with one another in space and time more often than with other conspecifics: [4]). Early SEMs emphasized the importance of food availability and predation risk [5–8], whereas later models added factors such as infanticide risk, pathogen transmission and information sharing [9–11]. SEMs typically focus on how one or more factors influence the emergence of the social relationships that are typical of each species (e.g. resident-nepotistic, dispersal-egalitarian [9]), rather than on explaining variation among social relationships within each group.

Recently, there has been growing interest in quantifying social complexity to test its role as a driving force in the evolution of communicative, cooperative and cognitive abilities [12–15]. There is a general consensus across disciplines that complexity emerges from the diverse interactions of various simpler elements, generating nonlinear effects that cannot be derived from the simpler elements on their own [15]. For example, the coordinated movements of bird flocks are interpreted as complex emergent properties of simple individual actions [16]. Social complexity can be viewed similarly, as emerging from the consistent variation in the frequency, patterning and types of social interactions that individual group members exchange with one another [17]. One way to assess social complexity is therefore by focusing on the number of differentiated relationships that individuals maintain with other group members, with a greater number of differentiated social relationships indicating greater social complexity [3,18]. In contrast with widely used proxies of social complexity such as group size, relationship differentiation takes the individual's perspective and focuses on how much social complexity an individual experiences within their group [19].

Socio-ecological factors are likely to influence social complexity, but no attempt has been made to apply SEMs to explain the differentiation of social relationships. Here, we propose a conceptual framework to shift the use of socio-ecological factors from characterizing typical social relationships for each species to explaining the extent of differentiation of social relationships within groups. Such differentiation ranges from individuals exhibiting similar frequencies, patterning and types of interactions with all other group members (i.e. low differentiation) to individuals exhibiting great variation in the frequency, patterning and/or types of interactions across group members (i.e. high differentiation). Most species are likely to be characterized by a degree of differentiation that is intermediate between these two extremes. For example, individuals may distinguish between kin or a subset of close associates, with whom they exchange high frequencies of affiliative interactions, and all other group members, with whom they exchange fewer and more ambivalent social interactions [3].

Our goal is to integrate the effects of multiple socio-ecological factors on relationship differentiation into a conceptual framework. To do so, we identify four components underlying multiple socio-ecological factors that influence the differentiation of social relationships: the extent of within-group contest competition to access resources (Component 1), the extent to which individuals differ in their ability to provide a variety of needed services (Component 2), the need for group-level cooperation (Component 3) and the constraints on social interactions (Component 4). We predict that Components 1 and 2 are the two major drivers of relationship differentiation, since at higher levels of each component, individuals are expected to receive direct fitness benefits by maintaining more differentiated relationships. By contrast, we expect components 3 and 4 to influence relationship differentiation primarily when at least one of the components 1 or 2 is high. We provide specific examples of how each of the four components is expected to impact on relationship differentiation. We then combine the four components into one framework and make predictions about the degree of relationship differentiation that can be expected according to the cumulative contribution of multiple socio-ecological factors to each of the four components.

## 2. Four components underlying socio-ecological factors that affect the differentiation of social relationships

### (a) Component 1: the extent of within-group contest competition to access resources

Within-group contest competition is expected to occur whenever resources can be monopolized [20]. Evidence suggests that a greater degree of within-group contest competition favours more despotic and nepotistic dominance hierarchies [9], thus driving social relationships towards greater differentiation. For example, high within-group contest competition for food resources should promote individual strategies to cooperate with a subset of preferred partners, such as kin, to monopolize access to limited food [6], resulting in some subsets of individuals with stronger relationships than other subsets. A comparison between two closely related species of squirrel monkeys with similarities in group size and diet nicely illustrates this example [21]. *Saimiri oerstedi* females rely heavily on smaller food patches that are not worth defending and exhibit mostly undifferentiated relationships with one another. By contrast, *S. sciureus* females form coalitions to defend access to larger food patches against other group members and show highly differentiated social relationships, which are stronger between coalition partners than between others [21].

Reproduction is another key resource for which within-group contest competition occurs. For example, males may compete for access to fertilization opportunities (e.g. [22,23]), whereas females may compete for access to preferred partners for parental investment and/or protection [24]. Although fertilization opportunities are less shareable resources than food, males may form intra-sexual alliances to gain access to fertile females, as occurs in bottlenose dolphins (*Tursiops truncatus*) and Barbary macaques (*Macaca sylvanus*) [25,26]. Similarly, females may form inter- or intra-sexual alliances to protect themselves and their offspring from male harassment [27,28]. Individuals may also compete for preferred spatial positions within the group, especially when predation risk is high, since individuals in the centre of the group have a lower probability of suffering predator attacks compared to individuals at the periphery [29,30]. Competition for preferred spatial positions may promote differentiation of social relationships whenever individuals are able to better monopolize access to this limited resource by cooperating within alliances.

### (b) Component 2: the extent to which individuals differ in their ability to provide a variety of needed services

Relationship differentiation can also occur due to differences in individual abilities to provide services, such as tolerance during feeding [31,32], coalitionary support [33,34], protection from harassment [35,36] or access to important information [37,38]. For example, some group members may be more effective than others in deterring predators, due to sexual dimorphisms or other traits [39]. Additionally, some individuals may be more effective coalition partners, due to their dominance rank or extent of shared interests [32–36]. Older, long-term residents are likely to be important sources of information about rare or ephemeral resources [40,41], whereas individuals of similar age and sex classes are more likely to possess the most relevant information pertaining to specific nutritional needs [42]. If all individuals have similar abilities to provide services, a low degree of relationship differentiation is expected. If many individuals can provide needed services, but some are better providers than others, an intermediate degree of relationship differentiation is expected. Relationship differentiation is greatest when different individuals are best suited to provide different services. Experiments manipulating the identity of holders of critical information provide empirical evidence that inter-individual variation in the ability to provide services can influence relationship differentiation [43,44]. For example, a low-ranking female vervet monkey (*Chlorocebus aethiops*), who was trained to open a container and provide food to her entire group received more grooming from more individuals than before the training. When a second trained provider was added, the first provider received less grooming, showing how social interactions are fine-tuned to changes in the number of service providers [43].

### (c) Component 3: the need for group-level cooperation

The need for cooperation with many or all group members to face external threats, including other groups or predators, promotes social tolerance across the group in order to achieve collective action [45]. A greater need for group-level cooperation should thus limit the differentiation of social relationships. For example, when between-group contest competition is high, individuals benefit by cooperating with a large number of group members to defend critical resources, such as food and reproductive opportunities, from other groups [9,46]. Similarly, when all group members can assist in predator defence and many are needed for effective predator deterrence (e.g. [47,48]), a greater need for group-level cooperation should promote more tolerant social relationships and limit the extent of relationship differentiation. For example, in green woodhoopoes (*Phoeniculus purpureus*), groups that experience more between-group conflict also exhibit a more even distribution of allopreening across group members, due to subordinates receiving more preening from the dominant breeding pair [49]. The effect of group-level cooperation on relationship differentiation is not necessarily limited to cooperation against external threats, but may also occur in other contexts when many group members are needed for effective cooperation. For example, lionesses of the same pride participate in cooperative hunting and communal breeding and exhibit relatively undifferentiated social relationships [50].

### (d) Component 4: constraints on social interactions

Multiple socio-ecological factors can limit the frequency, patterning or types of interactions among group members. For example, although similarities in terms of nutritional needs may increase competition in some contexts, they may also cause individuals to coordinate their activities preferentially with other group members with similar needs (e.g. based on reproductive state: [51–53]; based on age: [54]). In species with a high degree of fission–fusion dynamics [55], such coordination of activities may lead to subgrouping based on similar needs for extended periods of time [56]. Individuals may also occupy different spatial positions within the group (e.g. the centre versus the periphery due to antipredator strategies, see above). In both scenarios, spatial assortment may lead to social assortment, i.e. individuals who share proximity may develop social preferences for each other, promoting relationship differentiation, when social relationships are beneficial to deal with specific socio-ecological pressures (see Components 1 and 2). Under these circumstances, the impact of component 4 on relationship differentiation may be relatively low if based on a simple similarity rule (i.e. same needs versus different needs) but can be greater when the nature of multiple needs is taken into account (e.g. similar needs with respect to proteins, but different needs with respect to predation).

The risk of pathogen transmission can also influence relationship differentiation through a reduction in the frequency or types of social interactions with sick individuals. Under a high risk of pathogen transmission, group members should actively avoid sick individuals when possible, as an adaptive response to reduce infection [57] and may practice social distancing by more generally limiting their social interactions to a few key partners [58,59] as a trade-off between the benefits of social interactions and the potential costs of socializing in an environment with high pathogen prevalence [11]. For example, healthy mice reduce social interactions with parasite-infected mice, but not with other healthy individuals [60]. This reduction in social interactions should promote relationship differentiation similarly to the process of spatial assortment due to a simple similarity rule (see above).

## 3. Integrating the four components into one conceptual framework

**Figure 1.**
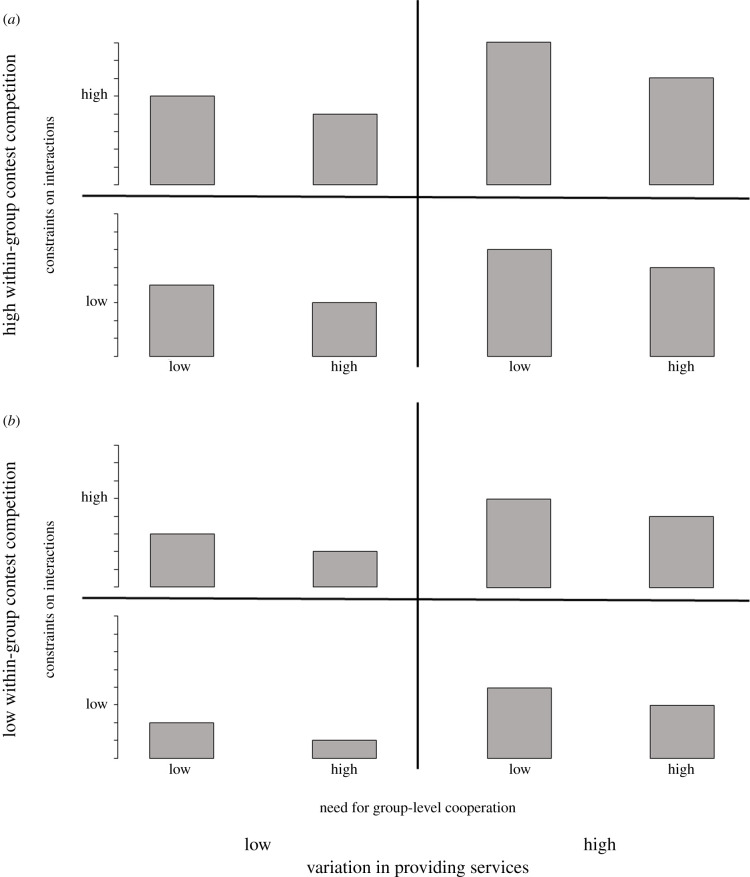
Graphical illustration of the conceptual framework integrating four components underlying socio-ecological factors. The height of the bars represents the extent of relationship differentiation resulting from the combinations of the four components. Although the level of each component varies along a continuous scale, each component is simply represented as high or low for ease of illustration. (*a*) and (*b*) illustrate the possible combinations of the other three components when the level of Component 1 (the extent of within-group contest competition to access resources) is high and low, respectively. For example, the top left bar in (*a*) represents the extent of relationship differentiation resulting from a high level of within-group contest competition, a low level of variation in the ability to provide services, a low need for group-level cooperation and many constraints on social interactions.

The degree of differentiation of social relationships within a group is not the result of one single factor but rather the consequence of multiple socio-ecological factors. Hence, we propose a conceptual framework integrating the effects of the four components outlined above, which can integrate the impacts of numerous socio-ecological factors, to either promote or discourage the differentiation of social relationships within groups. The contribution from multiple socio-ecological factors to each of the four components is cumulative, leading to synergistic or opposing effects on relationship differentiation. For example, when within-group contest competition to access resources (Component 1) is high for more than one socio-ecological factor (e.g. competition to access food, mates and safer positions), we predict that their synergistic effect in driving relationships toward greater differentiation is stronger than if contest competition is high for only one factor. Figure 1 summarizes the conceptual framework. We can use this framework to make predictions about the degree of differentiation of social relationships according to the combination of the four components. For example, the lowest bar in figure 1*b* illustrates the lowest degree of relationship differentiation, which we predict to occur when there is a low degree of within-group contest competition, little variation in the ability to provide services, high need for group-level cooperation and few constraints on social interactions. In this case, group members would have similar types and frequencies of interactions with one another, and social relationships should be mostly undifferentiated. By contrast, the highest bar in figure 1*a* illustrates the highest degree of relationship differentiation, which is expected to occur when there is a high degree of within-group contest competition, much variation in the ability to provide services, little need for group-level cooperation and many constraints on social interactions. In this case, we would expect highly differentiated social relationships because Components 1 and 2 (i.e. the two major drivers of relationship differentiation) are both high. The many constraints on social interactions would also promote relationship differentiation, whereas the limited need for group-level cooperation would not hamper relationship differentiation.

## 4. Conclusion

Competing theories of cognitive evolution emphasize the importance of either social or ecological challenges as the primary drivers of increased cognitive abilities (reviewed in: [61,62]). Here we proposed that ecological and social challenges are inter-related, via the cumulative impact of multiple socio-ecological factors on relationship differentiation. We did so by (i) focusing on four components underlying socio-ecological factors; (ii) predicting how influences from such factors on each component may either promote or discourage relationship differentiation and (iii) integrating the combined effects of these components into a novel conceptual framework. This framework has broad applicability, since the four components we introduced are likely to be relevant to a wide range of animal taxa and to additional socio-ecological factors not explicitly dealt with here. Hence, this framework promotes novel hypotheses about the cumulative impact of a variety of socio-ecological factors on the differentiation of social relationships within groups, as a proxy for variation in social complexity.

## Data accessibility

This article has no additional data.
